# Regional disparities and multidimensional determinants of hospital bed allocation in China: spatial evidence for public health policymaking

**DOI:** 10.3389/fpubh.2026.1813825

**Published:** 2026-05-07

**Authors:** Ke Hu, Xingjin Yang, Yu Cai, Chaojie Li, Xing Zhang, Di Xiao, Mingyang Yu

**Affiliations:** 1 Xiamen Haicang Hospital, Xiamen, Fujian, China; 2 QianDongNanZhou Center for Disease Control and Prevention, QianDongNanZhou, Guizhou, China; 3 Shanghai Municipal Hospital of Traditional Chinese Medicine, Shanghai, China; 4 Xingtai Center for Disease Control and Prevention, Xingtai, Hebei, China; 5 Nanjing Lishui Dongping Street Health Center, Nanjing, Jiangsu, China; 6 Community Health Service Center of Jiuxian Tongliang District, Chongqing, China; 7 Fuwai Central China Cardiovascular Hospital, Zhengzhou, Henan, China

**Keywords:** Geodetector, hospital beds, multiple linear regression, spatial autocorrelation, spatial error model

## Abstract

**Introduction:**

Medical resource distribution in China exhibits marked spatial disparities, with hospital bed allocation demonstrating particularly pronounced regional inequalities. These imbalances may significantly impact healthcare accessibility and population health outcomes across different provinces.

**Methods:**

This study employed a comprehensive analytical framework to examine hospital bed distribution across 31 provinces in China in 2022. We first conducted descriptive statistics and spatial autocorrelation analysis to identify distribution patterns. Subsequently, multiple linear regression (MLR) and spatial error model (SEM) were applied to evaluate the factors categorized into six dimensions: economic development, population structure, environmental conditions, education level, existing healthcare resources, and transportation infrastructure. Finally, the Geodetector method was systematically implemented to quantify individual factor contributions and their interaction effects based on spatial stratified heterogeneity principles.

**Results:**

Our analysis revealed three key findings: (1) Significant regional disparities existed in hospital beds per 1,000 population, with global spatial autocorrelation confirming strong clustering patterns. Local indicators of spatial association (LISA) identified high-high clusters in southwestern regions and low-low clusters in southeastern provinces; (2) Both MLR and SEM analyses indicated a negative correlation between sex ratio and bed availability, with SEM demonstrating superior goodness-of-fit by effectively addressing spatial autocorrelation; (3) Geodetector results showed GDP per capita served as the primary driver of bed distribution, revealing a threshold effect where bed demand concentrated in low-GDP regions. Notably, significant interaction effects that substantially magnified individual factor impacts, with the synergistic effect between disposable income per capita and sex ratio emerging as the most pronounced.

**Conclusion:**

This study reveals substantial spatial disparities in hospital bed distribution across China, highlighting the necessity of integrated regional policymaking that explicitly addresses multifactorial synergies to optimize equitable healthcare resource allocation.

## 1 Introduction

The spatial distribution of medical resources in China exhibits significant disparities, with hospital beds showing particularly pronounced regional inequalities. Research indicated that western provinces demonstrated the lowest equity in hospital bed allocation, displaying strong spatial clustering characteristics (1). This uneven distribution has created simultaneous resource shortages in some areas and underutilization in others. County-level analyses revealed that hospital bed density correlated positively with local savings levels and government fiscal revenue (1), suggesting an economically driven allocation pattern that exacerbates regional disparities.

Given this spatial heterogeneity, it is essential to analyze the multidimensional factors influencing bed allocation. For instance, economic factors (e.g., GDP per capita, disposable income) had been demonstrated to strongly correlate with bed density (2, 3). Similarly, demographic features (population density, sex ratio) significantly influenced bed numbers (4, 5), while healthcare resources indicators (e.g., physicians per 1,000 people) further contributed to spatial disparities (6). Moreover, transportation infrastructure played a critical role in shaping healthcare facility distribution (7). Additionally, education level was another key factor accounting for bed quantity differences (8). Notably, evidence suggested that PM_2.5_ indirectly elevated healthcare demand by increasing disease burden, thereby potentially straining bed occupancy rates (9). Collectively, these factors interacted through complex mechanisms, driving the observed spatial patterns.

Spatial regression models provide an effective solution to the issue of spatial dependence, which is often neglected in traditional statistical methods. The Spatial Lag Model (SLM) captures resource spillover effects between neighboring regions by incorporating a spatial lag term (10), while the Spatial Error Model (SEM) addresses spatial autocorrelation in residuals, thereby mitigating bias from unobserved spatial dependencies (11). Compared to conventional spatial regression approaches, Geodetector offers distinct methodological advantages. First, its *PD* (Power of Determinant) statistic quantifies the explanatory power of each variable on hospital bed distribution, enabling objective factor importance assessment (12). Second, through its interaction detection module, it identifies non-linear synergistic effects, such as the combined influence of economic development and population density on healthcare resource allocation (13). Finally, its assumption-free modeling framework is particularly suited for analyzing the non-linear relationships commonly observed public health data in China (14).

Existing studies have primarily examined individual provinces or specific resource types, with limited systematic analysis of multi-factor interactions at the national level. This study employed the Geodetector model to identify key drivers of bed distribution and their optimal ranges, combined with spatial regression modeling to analyze spatial dependence among influencing factors. By directly linking spatial equity metrics-such as regional clustering patterns and the identified drivers-to the core tenets of the “Healthy China 2030” initiative, which prioritizes equitable resource allocation and universal access to basic healthcare, this research provides a spatially nuanced evidence base. The findings provided a scientific basis for tailored regional healthcare policies, contributing to the equitable resource allocation goals of “Healthy China 2030”.

## 2 Methods

### 2.1 Data

Building upon the theoretical framework established in the background section and considering data availability, we selected eight key indicators for 2022 across six dimensions: economic development, population structure, environment condition, Education level, healthcare resources, and transportation infrastructure (see Table 1 for details).

**Table 1:** Key factors selected for analysis.

Categories	Factors	Data source
Economic development	GDP per capita	China statistical yearbook, 2023
	Disposable income per capita	
Population structure	Population density	
	Sex ratio	
Environment condition	PM_2.5_	Provincial environmental status reports, 2022
Education level	Illiteracy rate	China statistical yearbook, 2023
Healthcare resources	Number of licensed physicians per 1,000 population	
Transportation infrastructure	Number of operating buses	

#### 2.1.1 Economic development (GDP per capita and disposable income per capita)

Economic factors are closely associated with bed density. Within our spatial framework, we hypothesize that provinces with higher economic output and household purchasing power may allocate more resources to healthcare infrastructure, thereby increasing hospital bed availability and shaping its spatial clustering patterns.

#### 2.1.2 Population structure (population density and sex ratio)

Demographic features influence bed numbers. Population density serves as a proxy for the concentration of potential healthcare demand, with higher-density areas expected to require greater bed capacity. Sex ratio is included due to sex-specific differences in healthcare utilization and hospitalization rates, which may influence bed demand patterns across regions.

#### 2.1.3 Environmental exposure (PM2.5 concentration)

PM_2.5_ may elevate healthcare demand by increasing disease burden. We include PM_2.5_ as an environmental covariate based on its potential to increase disease burden and healthcare demand. In our spatial context, we hypothesize that regions with higher PM_2.5_ concentrations may experience elevated respiratory disease prevalence, thereby straining bed occupancy rates and influencing bed allocation patterns.

#### 2.1.4 Educational attainment (illiteracy rate)

Education level accounts for differences in bed quantity. Education serves as a proxy for health literacy, which influences healthcare-seeking behaviors and disease prevention awareness. For our spatial analysis, we posit that regional variations in illiteracy rates may lead to spatial disparities in healthcare utilization, thereby affecting the effective demand for hospital beds.

#### 2.1.5 Healthcare resources (number of licensed physicians per 1,000 population)

Healthcare resource indicators contribute to spatial disparities in bed allocation. The capacity of the healthcare workforce, as indicated by physician density, is expected to correlate positively with hospital bed supply, as regions with more robust healthcare infrastructure tend to allocate greater resources to inpatient care capacity.

#### 2.1.6 Transportation infrastructure (number of operating buses)

Transportation infrastructure shapes healthcare facility distribution by affecting population mobility and geographic accessibility to medical services. We hypothesize that regions with better-developed transportation networks may facilitate patient movement across regions, potentially influencing the spatial distribution of bed demand and utilization patterns.

The study was conducted at the provincial level, encompassing all 31 mainland Chinese administrative regions (excluding Hong Kong, Macao, and Taiwan).

The dependent variable (number of hospital beds per 1,000 population) was obtained from the 2023 China Health Statistical Yearbook, which reports data for the year 2022. PM_2.5_ concentration data were sourced from the 2022 provincial environmental status reports. All other independent variables were drawn from the 2023 China Statistical Yearbook, also covering the year 2022.

### 2.2 Descriptive analyses

The spatial distribution characteristics of hospital beds in the 31 districts were illustrated through thematic mapping.

### 2.3 Spatial autocorrelation analysis

This study employed two fundamental spatial autocorrelation measures: Global Moran's *I* and Local Moran's *I* indices.

#### 2.3.1 Global Moran's I Index

The Global Moran's *I* statistic is utilized to assess the overall spatial autocorrelation pattern across the study area (15), with values ranging from −1 to 1.

Its mathematical formulation is as follows (Equation 1) (16):


$$\begin{array}{l} I\text{=}\frac{n\overset{n}{\underset{i=1}{\sum }}\overset{n}{\underset{j=1}{\sum }}W_{ij}\left(x_{i}-\bar{x}\right)\left(x_{j}-\bar{x}\right)}{\overset{n}{\underset{i=1}{\sum }}\overset{n}{\underset{j=1}{\sum }}W_{ij}\overset{n}{\underset{i=1}{\sum }}{\left(x_{i}-\bar{x}\right)}^{2}} \end{array}$$ (Equation 1)

The Global Moran's *I* index ranges from −1 to 1, where n represents the sample size, *W*_ij_ denotes the elements of the spatial weight matrix, *x*_i_ and *x*_j_ are the observed values, and $\bar{x}$ is the mean value.

A significantly positive value (*p* < 0.05) indicates spatial clustering of similar values, while a negative value suggests spatial dispersion (17).

#### 2.3.2 Local Moran's Index (LISA) analysis

The LISA are employed to identify localized spatial heterogeneity patterns. The Local Moran's *I* statistic for each spatial unit was calculated as Equation 2 (18):


$$\begin{array}{l} I_{i}=\frac{n\left(x_{i}-\bar{x}\right)\overset{n}{\underset{j=1}{\sum }}w_{ij}\left(n_{j}-\bar{n}\right)}{\overset{n}{\underset{j=1}{\sum }}{\left(x_{j}-\bar{x}\right)}^{2}} \end{array}$$ (Equation 2)

The Local Moran's *I* (LISA) employs the same variable definitions as the Global Moran's *I* to identify four characteristic spatial association patterns: high-high clusters (HH), low-low clusters (LL), high-low outliers (HL), and low-high outliers (LH). These spatial patterns are statistically verified through significance testing and visually represented using LISA cluster maps to demonstrate the spatial heterogeneity (19).

### 2.4 Multiple linear regression (MLR)

MLR analysis is employed to examine the linear relationships between multiple independent variables and the dependent variable (20), expressed as Equation 3:


$$\begin{array}{l} Y=\beta_{0}+\beta_{1}X_{1}+\beta_{2}X_{2}+...+\beta_{p}X_{p}+\varepsilon \end{array}$$ (Equation 3)

Where *Y* represents the dependent variable, *X*_1_ to *X*_p_ denote independent variables, β_0_ is the intercept, β_1_ to β_p_ are regression coefficients, and ε is the normally distributed error term with mean zero. Model performance is evaluated using the coefficient of determination (R^2^), AIC, and log-likelihood values, while multicollinearity among factors is assessed through variance inflation factors (VIF).Variables with VIF >5 may exhibit multicollinearity (21).

### 2.5 Spatial regression models

#### 2.5.1 Spatial error model (SEM)

The SEM addresses spatial autocorrelation in regression residuals (11), formulated as Equations 4, 5:


$$\begin{array}{l} Y\;=\;X_{\beta }+\;u \end{array}$$ (Equation 4)

$$\begin{array}{l} u\;=\;\lambda W_{u}+\;\varepsilon \end{array}$$ (Equation 5)

Here, λ denotes the spatial error coefficient and *W*_u_ represents the spatially lagged error term. SEM effectively mitigates estimation bias caused by residual spatial dependence through incorporating a spatial error structure (22). Parameters are estimated via maximum likelihood, with residual spatial autocorrelation evaluated using Moran's *I* test.

#### 2.5.2 Spatial lag model (SLM)

The SLM captures spatial dependence by incorporating a spatially lagged dependent variable (Equation 6) (11):


$$\begin{array}{l} Y\;=\;\rho W_{Y}+\;X_{\beta }+\;\varepsilon \end{array}$$ (Equation 6)

Where ρ is the spatial autoregressive coefficient and *W*_Y_ denotes the spatially lagged dependent variable. This model is particularly suitable for analyzing spatial spillover effects between variables.

### 2.6 Geodetector analysis

Unlike conventional regression models, the Geodetector method does not assume independence among predictors and is inherently robust to multicollinearity. Consequently, correlated variables including those excluded from regression models due to high VIFs can be legitimately included to evaluate their individual and interactive explanatory power. The Geodetector method is a spatial statistical approach based on the principle of stratified heterogeneity. This method systematically analyzes spatial differentiation patterns and their driving mechanisms through four functional modules: (1) factor detector (quantifying explanatory power using *PD*-statistic), (2) interaction detector (examining factor synergies), (3) risk detector (identifying high-risk zones), and (4) ecological detector (testing significance of factor differences) (23–25).

#### 2.6.1 Factor detector

The factor detector module quantifies the explanatory power of individual variables through the *PD*-statistic, which compares within-stratum variance to total variance. This core algorithm precisely measures independent contribution of each factor to observed spatial heterogeneity patterns (Equation 7) (26).


$$\begin{array}{l} PD=1-\frac{\overset{L}{\underset{h=1}{\sum }}N_{h}\sigma_{h}^{2}}{N\sigma^{2}} \end{array}$$ (Equation 7)

The statistical parameters include *N*_h_ (sample size of stratum *h*), σ_h_^2^ (variance of stratum *h*), *N* (total sample size), and σ^2^ (total variance), with the *PD* value (also called *q*-statistic) ranging between 0 and 1, where higher values indicate greater explanatory power of the factor for spatial differentiation.

#### 2.6.2 Risk detector

The risk detector module statistically evaluates spatial risk level differences across factor stratifications using independent samples *t*-tests (23). It compares mean values between strata to identify significant high/low risk areas (Equation 8).


$$\begin{array}{l} t_{{ȳ}_{h-1}-{ȳ}_{h-2}}=\frac{{Ȳ}_{h=1}-{Ȳ}_{h=2}}{{\left[\frac{\mathrm{Var}\left({Ȳ}_{h=1}\right)}{n_{h=1}}+\frac{\mathrm{Var}\left({Ȳ}_{h=2}\right)}{n_{h=2}}\right]}^{1/2}} \end{array}$$ (Equation 8)

Where Ȳ*h*represents the average hospital beds of layer *h*, *n*_h_ is sample, var represents sample variance, *t* follows the Student's-*t*-test distribution.

The null hypothesis as follows (Equation 9);


$$\begin{array}{l} H_{0}:{Ȳ}_{h=1}={Ȳ}_{h=2} \end{array}$$ (Equation 9)

The test statistic follows a Student's *t*-distribution under the null hypothesis. Rejection of *H*_0_ at significance level α indicates statistically significant hospital beds differences between regions.

#### 2.6.3 Ecological detector

The ecological detector module assesses whether different factors exhibit statistically significant differences in their explanatory power for observed spatial patterns. Using ANOVA principles, it computes an *F*-statistic to compare within-stratum variances between factors (Equations 10–12):


$$\begin{array}{l} F=\frac{n_{X1}\left(n_{x2}-1\right)SSW_{X1}}{n_{X2}\left(n_{x1}-1\right)SSW_{X2}} \end{array}$$ (Equation 10)

$$\begin{array}{l} SSW_{X1}=\overset{L_{1}}{\underset{h=1}{\sum }}N_{h}\sigma_{h}^{2} \end{array}$$ (Equation 11)

$$\begin{array}{l} SSW_{X2}=\overset{L_{2}}{\underset{h=1}{\sum }}N_{h}\sigma_{h}^{2} \end{array}$$ (Equation 12)

Where *n*_x1_ and *n*_x2_ represent the samples of two factors *x*_1_ and *x*_2_, respectively. *SSW*_x1_ and *SSW*_x2_ represent the sum of the within-strata variance of *x*_1_ and *x*_2_, respectively; *L*_1_ and *L*_2_ represent the number of layers of *x*_1_ and *x*_2_, respectively.

The null hypothesis *H*_0_ posits no significant difference in explanatory power between factors. Rejection of *H*_0_ indicates statistically significant differences in factors capacity to explain spatial heterogeneity.

#### 2.6.4 Interaction detector

The interaction detector is primarily employed to analyze the mechanisms by which two-factor interactions influence the spatial differentiation of geographical phenomena. It identifies five interaction types (14): non-linear-weakening, univariate-weakening, bivariate enhancement, independent, and non-linear-enhancement effects (Table 2).

**Table 2:** Interaction types between variables.

Description	Interaction
*PD*(*X*_1_∩*X*_2_) < Min(*PD*(*X*_1_), *PD*(*X*_2_))	Weaken, non-linear
Min(*PD*(*X*_1_), *PD*(*X*_2_)) < *PD*(*X*_1_∩*X*_2_) < Max(*PD*(*X*_1_)), *PD*(*X*_2_))	Weaken, univariate
*PD*(*X*_1_∩*X*_2_)>Max(*PD*(*X*_1_), *PD*(*X*_2_))	Enhanced, bivariate
*PD*(*X*_1_∩*X*_2_) = *PD*(*X*_1_)+*PD*(*X*_2_)	Independent
*PD*(*X*_1_∩*X*_2_)>*PD*(*X*_1_)+*PD*(*X*_2_)	Enhance, non-linear

#### 2.6.5 Variable discretization methods

For Geodetector analysis, continuous variables require discretization into categorical data. Five standard methods were evaluated (27):

(1) Natural breaks classification

This data-driven approach automatically identifies optimal classification boundaries by maximizing inter-class variance while minimizing intra-class variance, based on the inherent distribution characteristics of the dataset.

(2) Quantile classification method

This non-parametric classification scheme ensures an equal number of observations in each category, making it particularly suitable for datasets exhibiting skewed or non-uniform distributions.

(3) Equal interval classification

The value range is partitioned into intervals of equal width, providing optimal performance when analyzing uniformly distributed variables.

(4) Geometric interval classification

Class boundaries are determined using a geometric progression series, which effectively handles data following exponential distribution patterns.

(5) Standard deviation classification

Centered around the mean value, this method establishes classification thresholds at integer multiples of the standard deviation, specifically designed for normally distributed data.

The optimal discretization strategy was selected following the principle proposed by Cao et al. (27), which recommends choosing the classification scheme that maximizes the *PD* value. Accordingly, we systematically evaluated five discretization methods and a range of class intervals (typically three to eight, depending on the method) for each independent variable, selecting the scheme that maximized the *PD* value.

### 2.7 Software implementation

Spatial analyses were conducted using ArcGIS 10.2 (Discretization, Spatial Autocorrelation Analysis, Mutlicollinearity diagnostics, Visualization) and GeoDa 1.22 (MLR,SEM,SLM). Geodetector analysis used the Excel version (http://Geodetector.cn). All maps were sourced from the National Geospatial Information Platform [Authorization GS(2024)0650]. Statistical significance was assessed at *p* < 0.05 (two-tailed).

## 3 Results

### 3.1 Spatial distribution characteristic and aggregation characteristics of hospital beds

**Figure 1:**
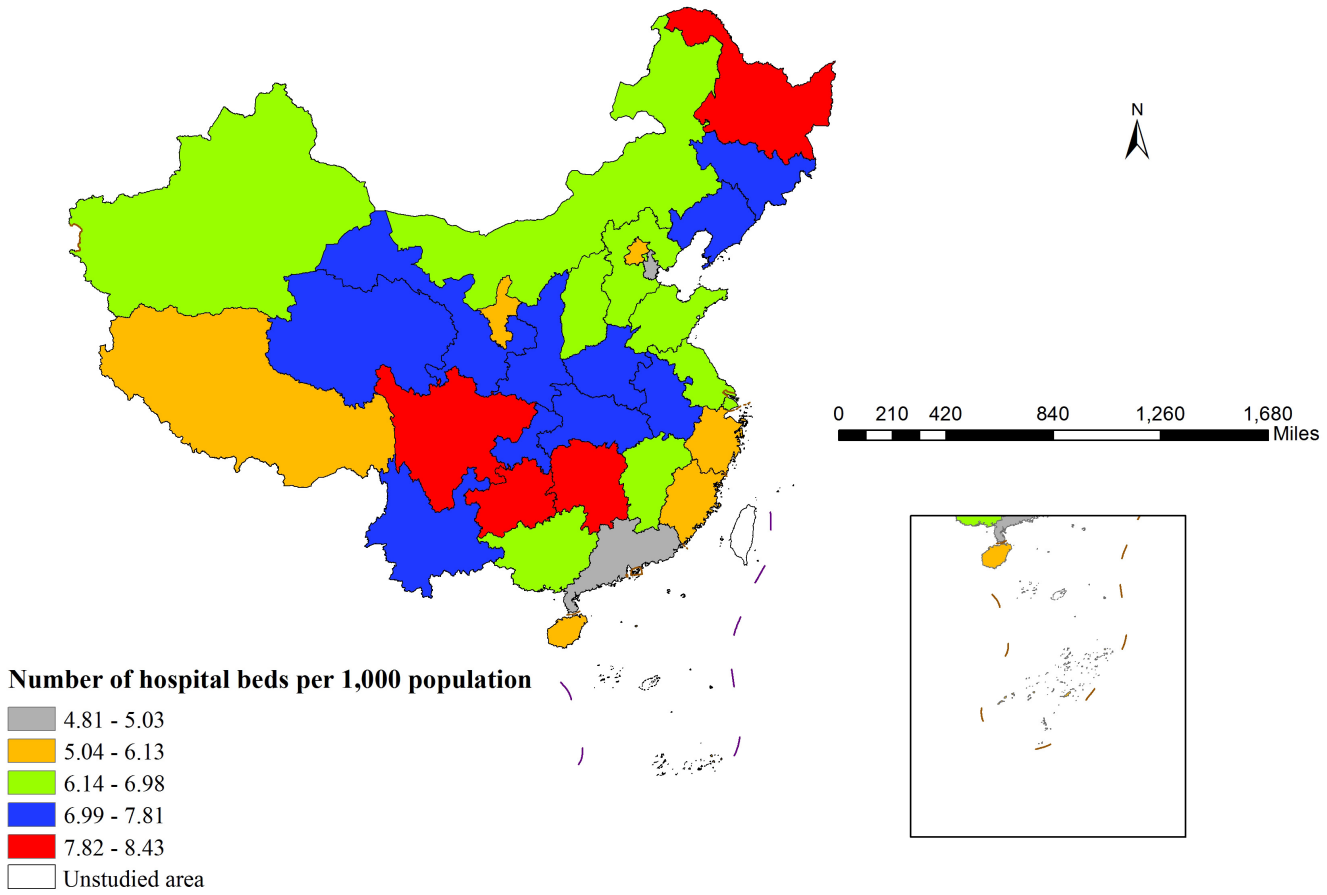
Spatial distribution of hospital beds per 1,000 population across China in 2022.

In 2022, marked regional disparities in hospital beds per 1,000 population were observed across China (as shown in Figure 1). Heilongjiang recorded the highest density (8.43), whereas Tianjin reported the lowest (5.03). Geographically, southwestern and northeastern regions generally exhibited higher bed availability than their southeastern counterparts.

**Figure 2:**
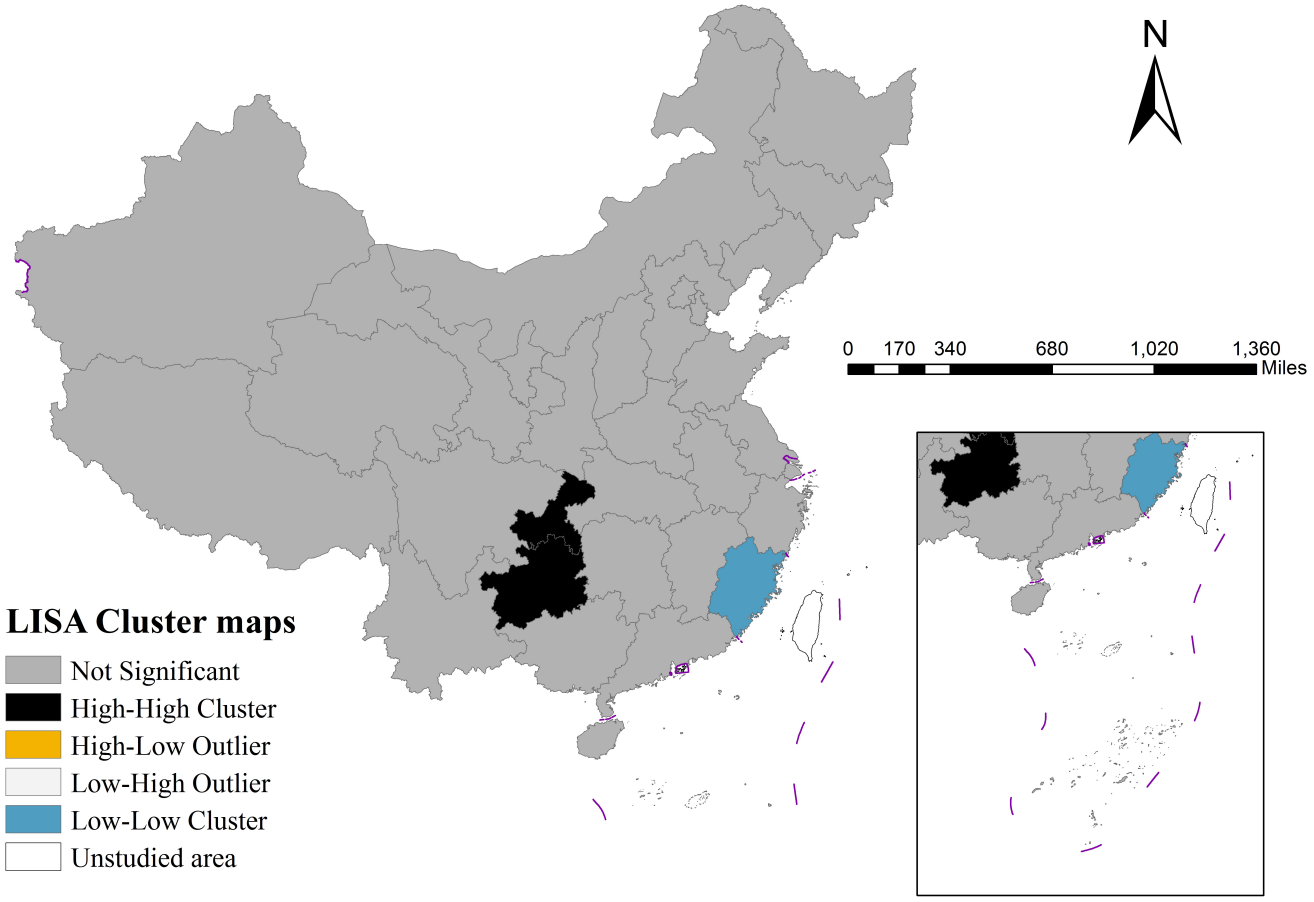
LISA cluster map of hosptial beds in China, 2022.

Global spatial autocorrelation analysis detected significant spatial clustering (Moran's *I* = 0.277, *p* = 0.008). As illustrated in Figure 2, LISA cluster mapping revealed two pre-dominant patterns: high-high clusters concentrated in southwestern regions (e.g., Guizhou and Chongqing) and low-low clusters in southeastern provinces (e.g., Fujian).

### 3.2 Regression analysis

Initial multicollinearity diagnostics showed excessive VIF values for GDP per capita (11.893) and disposable income per capita (28.621). Given that VIF values exceeding five are commonly interpreted as indicating severe multicollinearity, and considering that removing both variables would leave the economic development dimension without any representative indicator, we removed disposable income per capita-the variable with the higher VIF-while retaining GDP per capita. Theoretically, GDP per capita serves as a comprehensive indicator of regional economic development and directly reflects local governments' fiscal capacity for healthcare infrastructure investment. In China's context, where hospital bed allocation is pre-dominantly shaped by government-led planning and public expenditure, GDP per capita is more directly relevant to the supply-side availability of beds. In contrast, disposable income per capita primarily captures household purchasing power and is more closely associated with healthcare utilization rather than the supply of hospital beds, which is the focus of this study. While disposable income per capita may be a more direct driver of healthcare demand at the household level, the present study aims to explain the spatial distribution of bed supply, for which GDP per capita serves as a more appropriate indicator of the fiscal capacity underpinning infrastructure investment. Thus, retaining GDP per capita aligns with our analytical emphasis on the determinants of bed supply. Following this adjustment, all remaining VIF values fell below 5, with detailed VIF results presented in Table 3. The MLR model identified only sex ratio as statistically significant (Table 3). However, Moran's *I* coefficient of residuals demonstrated significant spatial autocorrelation (*p* < 0.05), indicating the necessity of employing spatial regression models for further analysis.

**Table 3:** Results of the SEM model and MLR model.

Factors	SEM		MLR		VIF
	Coefficient	*p*-value	Coefficient	*p*-value	
Intercept	22.3553	0.0000	25.1048	0.0005	/
GDP per capita	−0.0000	0.2768	−0.0000	0.3559	4.204
Population density	0.0002	0.3965	0.0002	0.4997	2.342
Sex ratio	−0.1478	0.0005	−0.1531	0.0082	1.909
PM_2.5_	0.0016	0.9393	−0.0022	0.9223	1.862
Illiteracy rate	−0.0351	0.1276	−0.0259	0.3787	1.514
Number of licensed physicians per 1,000 population	0.2000	0.5686	−0.4352	0.2933	2.147
Number of operating buses	−0.0000	0.5665	−0.0000	0.6543	1.302
*R^2^*	0.6028		0.5074		/
AIC	74.8170		78.0925		
Log Likelihood	−29.4085		−31.0463		
Moran's *I*	0.0110	0.3210	0.1105	0.0335	

The selection between SLM and SEM was guided by both theoretical considerations and empirical performance. Theoretically, SEM is more appropriate when spatial autocorrelation originates from unobserved spatially correlated factors-such as shared regional health policies, cultural norms of healthcare-seeking behavior, or unmeasured environmental characteristics-rather than from direct spillover effects of the dependent variable across neighboring regions. Given that hospital bed allocation in China is primarily determined by centralized regional planning and policy frameworks rather than interprovincial spillover of bed density itself, we theoretically prioritized SEM as the more suitable specification. The spatial regression analysis compared the performance of SLM and SEM. The SLM showed a non-significant spatial lag coefficient (ρ = 0.064, *p* = 0.568), while the SEM demonstrated a highly significant spatial error coefficient (λ = 0.626, *p* < 0.001), empirically validating our theoretical reasoning. The SEM results (Table 3) revealed that only sex ratio significantly influenced bed distribution, with stronger significance than the MLR model (*p* = 0.0005 vs. 0.0082). Specifically, the SEM coefficient for sex ratio was −0.1478 (*p* = 0.0005), indicating that for every one-unit (one percentage point) increase in the sex ratio (i.e., a greater proportion of males relative to females), the number of hospital beds per 1,000 population decreased by approximately 0.15 beds, holding other factors constant. The SEM exhibited superior model fit, evidenced by higher R^2^ and log-likelihood values, lower AIC, and non-significant residual spatial autocorrelation (Moran's *I*), confirming its effectiveness in accounting for spatial effects.

### 3.3 Results of the Geodetector model

#### 3.3.1 Discretization of independent variables

To ensure optimal classification of the eight independent variables, five distinct discretization methods were employed: equal interval, natural breaks, quantile, geometric interval, and standard deviation classification. Each variable was categorized into 3–8 classes where feasible, and the optimal classification scheme was determined by calculating the *PD* (power of determinant) values using the factor detector module of Geodetector. The classification method and interval yielding the highest *PD* value were selected as the optimal approach. Detailed discretization results are presented in Table 4. A comprehensive comparison of *PD*-values across the five discretization methods and various class intervals for each factor, along with the selected optimal parameters, is provided in Supplementary Table S1 (Comparison of *PD*-values for each factor under different discretization strategies and selection of optimal parameters).

**Table 4:** Results of factor detection and variable classification.

Factors	*PD*	*p*	Classification method	Classification interval
GDP per capita	0.57	0.029	Quantile	8
Disposable income per capita	0.51	0.026	Geometrical interval	6
Population density	0.30	0.037	Quantile	4
Sex ratio	0.50	0.047	Quantile	8
PM_2.5_	0.41	0.664	Quantile	7
Illiteracy rate	0.25	0.597	Quantile	8
Number of licensed physicians per 1,000 population	0.36	0.212	Quantile	8
Number of operating buses	0.35	1.000	Equal interval	8

#### 3.3.2 Factor detector analysis

As illustrated in Table 4, the influence of PM_2.5_ concentration, illiteracy rate, number of physicians per thousand population, and number of operating buses on hospital bed availability was statistically insignificant (*p* > 0.05). In contrast, the remaining factors exhibited *PD* values ranging from 0.3 to 0.57, indicating varying degrees of explanatory power. Notably, GDP per capita demonstrated the strongest association (*PD* = 0.57), while population density exhibited a relatively weaker influence (*PD* = 0.3).

#### 3.3.3 Risk detector findings

**Figure 3:**
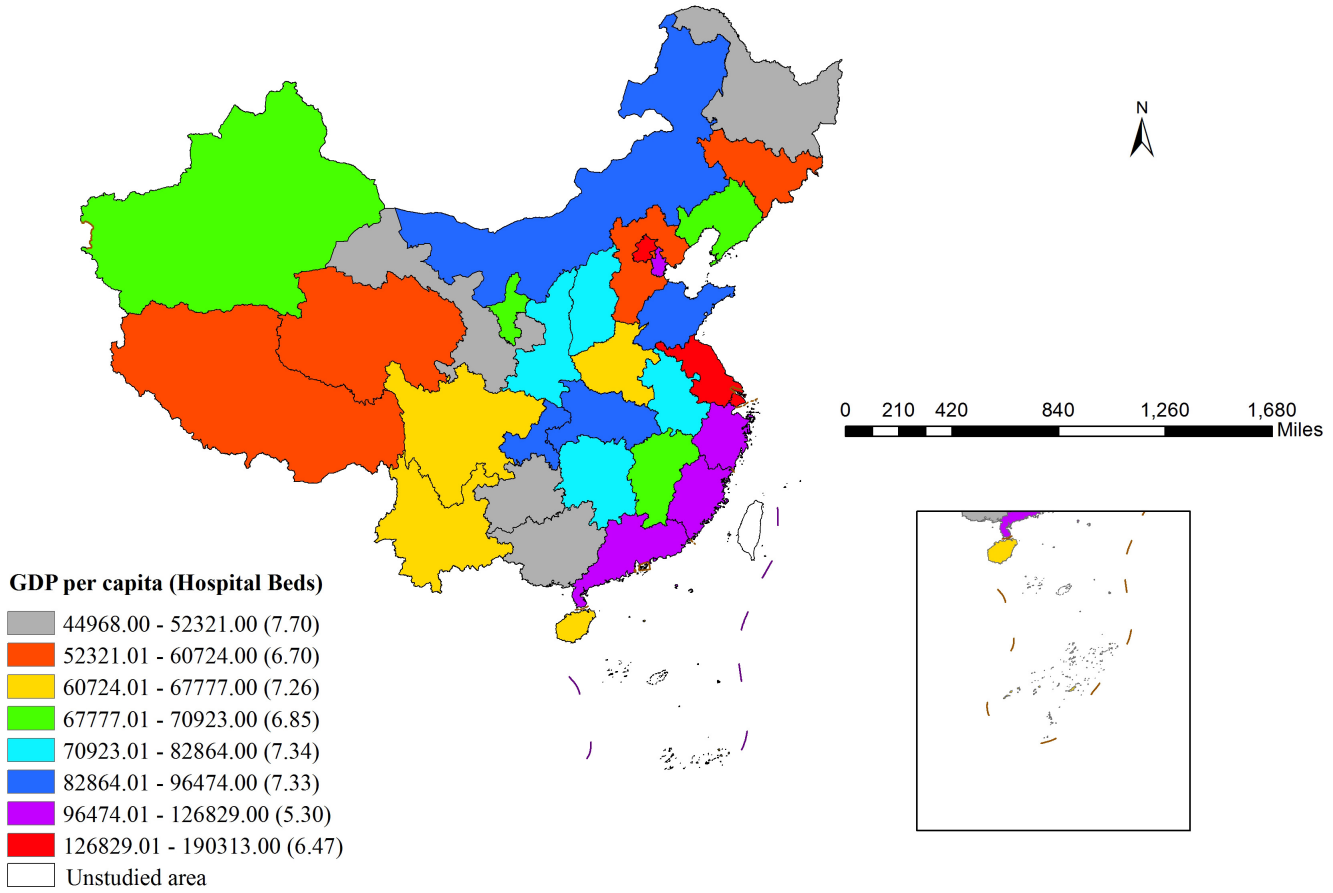
Regional disparities in GDP per capita and associated hospital beds.

The risk detector module facilitated the assessment of hospital beds across different strata of each determinant while evaluating inter-strata differences. Figure 3 presents the non-linear relationship between GDP per capita and hospital bed availability. Bed counts reached their minimum (5.30) when GDP per capita fell within 96,474.01–126,829.00 RMB, whereas the peak value (7.70) occurred in the lowest GDP range of 44,968–52,321 RMB, indicating a threshold effect whereby lower-GDP regions concentrate bed resources while higher-GDP regions exhibit fewer beds.

Table 5 summarizes the statistical significance of bed count variations across GDP per capita strata (Y: significant; N: non-significant). The 8th stratum (126,829.01–190,313.00 RMB) and 1st stratum (44,968–52,321 RMB) represented the highest and lowest GDP ranges, respectively. Notably, the 7th stratum showed statistically significant differences in bed counts compared to most other strata.

**Table 5:** Significance of differences in mean hospital beds across GDP per capita strata.

Stratum	1	2	3	4	5	6	7	8
1								
2	N							
3	N	N						
4	N	N	N					
5	N	N	N	N				
6	N	N	N	N	N			
7	Y	N	Y	Y	Y	Y		
8	Y	N	N	N	N	Y	Y	

**Figure 4:**
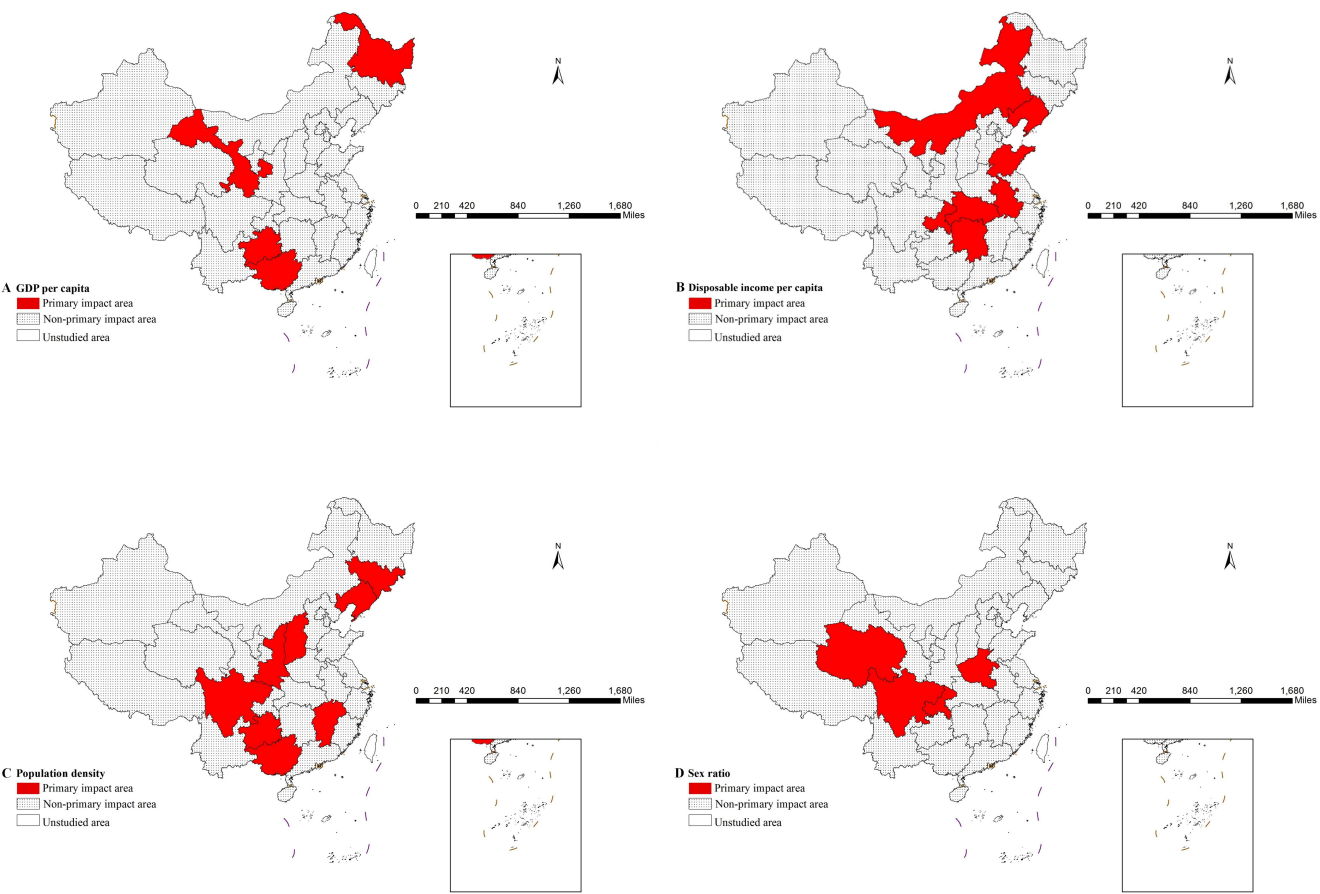
Distribution of main impact area of each factors **(A)** GDP per capita **(B)** disposable income per capita **(C)** population density **(D)** sex ratio.

Furthermore, the risk detector enabled quantitative analysis between determinants and bed availability by identifying optimal value ranges corresponding to peak bed counts (Table 6) (28). Notably, regions with the highest number of hospital beds can be considered as the primary impact area for each factor (29), with results visually presented in Figures 4A–D.

**Table 6:** Optimal range of factors and their corresponding peak hospital beds.

Factors	Optimal range	Number of hospital beds per 1,000 persons
GDP per capita (RMB)	44,968–52,321	7.70
Disposable income per capita (RMB)	32,709.63–39,856.10	7.46
Population density (persons/km^2^)	122.48–282.25	7.39
Sex ratio (%)	99.97–102.04	7.71

#### 3.3.4 Ecological detector results

The ecological detector was employed to conduct a comparative analysis of the potential differences (*PD* values) between any two key determinants of hospital bed capacity, assessing the statistical significance of their differential impacts (Table 7). The results demonstrated that only the *PD* value difference between GDP per capita and population density reached statistical significance (Y = significant, N = non-significant).

**Table 7:** Significance testing for *PD* value differences between factors.

Factors	GDP per capita	Disposable income per capita	Population density	Sex ratio
GDP per capita				
Disposable income per capita	N			
Population density	Y	N		
Sex ratio	N	N	N	

These findings suggested that among the key determinants influencing hospital bed capacity, GDP per capita and population density exhibited significantly distinct effects. This may indicate that these two variables possess unique explanatory power in determining bed availability. No statistically significant differences were observed between other key determinants. To further elucidate the complex interactions among these factors and their combined effects on hospital bed capacity, interaction detector analysis was subsequently performed.

#### 3.3.5 Interaction detector results

The interaction detector analysis reveals significant interactive effects between examined factors and characterizes their specific effect types. As shown in Table 8, the *PD* values of all two-factor interactions exceeded those of individual factors, with minimum post-interaction *PD* values consistently above 0.7. Among all interactions, the combination of disposable income per capita and sex ratio yielded the highest *PD* value (0.97). Further examination of interaction types identified a non-linear enhancement effect between population density and sex ratio, while other variable combinations exhibited bivariate enhancement effects.

**Table 8:** Interaction effects between risk factors on hospital beds.

Factors	GDP per capita	Disposable income per capita	Population density	Sex ratio
GDP per capita	0.57			
Disposable income per capita	0.71	0.51		
Population density	0.77	0.70	0.30	
Sex ratio	0.93	0.97	0.88	0.50

## 4 Discussion

Our modeling results demonstrated significant improvements after employing the SEM. The influence of sex ratio showed enhanced statistical significance, while the model's goodness-of-fit substantially improved with eliminated residual spatial autocorrelation, confirming effectiveness of SEM in addressing spatial dependency issues. These findings align with existing literature indicating that SEM provides more reliable parameter estimates and statistical inferences than conventional regression methods when spatial correlation exists in the data (22, 30).

Both the MLR and SEM identified sex ratio as the only statistically significant factor negatively associated with hospital bed availability-a finding that contrasts with the broader literature emphasizing economic and demographic drivers. This divergence warrants explicit interpretation. The negative effect of sex ratio on bed availability may stem from the following reasons: In low- and middle-income countries, female patients have a significantly lower probability of receiving surgical treatment compared to males (31). This disparity in care may lead to shorter hospital stays or reduced hospitalization rates among women, thereby decreasing the actual demand for beds and manifesting as a negative regulatory effect of sex ratio on bed availability. Additionally, males exhibited higher hospitalization rates for certain diseases (e.g., trauma, respiratory conditions) (32), while females might occupy different types of beds due to sociocultural factors (e.g., maternal care needs). When the sex ratio was imbalanced (e.g., an overrepresentation of males), it might intensify bed pressure in specific departments (e.g., surgery) (33), while resources in other departments (e.g., obstetrics and gynecology) remained underutilized, resulting in an overall decline in efficiency.

The non-significance of other variables in the two models warrants separate interpretation. In MLR, the significant residual spatial autocorrelation (Moran's *I* = 0.1105, *p* = 0.0335) indicates that unaddressed spatial dependence violates the independence assumption, leading to inefficient estimates and inflated standard errors that obscure the effects of other factors. In contrast, the SEM explicitly accounts for spatial error dependence (λ = 0.626, *p* < 0.001). The non-significance of GDP per capita, population density, and physician density in the SEM may be explained by several interconnected mechanisms. First, unobserved spatial structures-such as regional healthcare planning policies or interprovincial resource sharing mechanisms-may mediate the effects of conventional socioeconomic factors, thereby attenuating their direct statistical significance. Second, the provincial level of analysis may mask sub-provincial variations; factors such as GDP per capita or physician density may exert stronger influences at the city or county level, where resource allocation decisions are often operationalized. Third, the inclusion of spatial effects in the SEM may have absorbed part of the explanatory power traditionally attributed to these factors. Collectively, these considerations suggest that the absence of significant coefficients for these factors does not negate their substantive importance but rather highlights the necessity of accounting for spatial dependency and scale effects when interpreting drivers of healthcare resource distribution.

Notably, in contrast to the SEM, the Geodetector Model successfully identified four key determinants significantly influencing bed availability. This divergence reflects fundamental methodological differences: the SEM assumes linearity and estimates average marginal effects across space, whereas Geodetector is a non-parametric approach designed to detect spatial stratified heterogeneity without assuming linear relationships. Consequently, the non-significant coefficient for GDP per capita in the SEM does not contradict its identification as a key driver in Geodetector, but rather suggests that its relationship with bed distribution may be non-linear-a pattern the Geodetector is specifically suited to uncover. This innovative approach employs *PD*-statistics to quantify the relative contribution of each factor, while inherently addressing multicollinearity issues. Such distinctive capability provides the Geodetector with superior advantages in examining spatially heterogeneous drivers, particularly when analyzing complex spatial datasets where conventional spatial regression approaches encounter methodological constraints.

The factor detector analysis demonstrated that GDP per capita exerted the most substantial influence on bed availability with a *PD* value of 0.57, whereas the risk detector surprisingly identified the highest bed numbers occurring at relatively lower GDP levels. As shown in Figure 3, bed counts peaked in the lowest GDP stratum (44,968–52,321 RMB) and reached their lowest point (5.30) in the 96,474.01–126,829.00 RMB stratum, indicating a threshold effect. This threshold is approximately 96,474 RMB per capita: below this level, bed availability ranges from 6.70 to 7.70 beds per 1,000 population; above this level, bed availability ranges from 5.30 to 6.47 beds, with the nadir (5.30) occurring immediately above the threshold. This non-linear pattern helps explain why GDP per capita was not statistically significant in the SEM, as spatial regression models assume linearity and may not capture such threshold-based relationships. This finding challenges conventional economic theories that assume a monotonic positive relationship between economic development and healthcare resource supply. Instead, it suggests a non-monotonic, threshold-based relationship, where beyond a certain level of development, further economic growth does not translate into more hospital beds but rather into efficiency gains and quality improvements. This apparent paradox can be explained through several interrelated mechanisms since regions with lower GDP tend to centralize medical resources to meet basic healthcare needs, consequently reaching peak bed availability (34). Moreover, when GDP surpasses a critical threshold, the improved efficiency in medical resource allocation diminishes the necessity for bed quantity expansion (34), while higher-GDP regions increasingly shift their focus toward quality enhancement rather than quantity growth by optimizing medical staff allocation and technological advancement instead of merely adding beds (35). Additionally, spatial factors such as population density generate negative spillover effects that effectively suppress bed concentration in economically advanced areas (36), and furthermore, developed regions place greater emphasis on preventive care and outpatient services, thereby significantly reducing their reliance on inpatient beds (37).

Furthermore, the factor detector results indicated no significant association between bed and PM_2.5_, illiteracy rates, physician density, or public transportation capacity (*p* > 0.05), suggesting these effects of factors may be mediated by other socioeconomic variables or require higher-resolution data for detection.

The interaction detector analysis revealed that interactive effects between factors significantly amplified their individual impacts, as evidenced by all interaction terms exhibiting higher *PD* values than single factors. Notably, the interaction between disposable income per capita and sex ratio demonstrated the most pronounced synergistic effect (*PD* = 0.97). The divergence in healthcare demands caused by sex ratio imbalances is amplified by higher incomes, while lower incomes worsen resource shortages, collectively exacerbating the bed supply-demand mismatch. Such enhancement effects underscore the necessity of accounting for combined socioeconomic and demographic influences when formulating public health policies (38).

This study integrates spatial regression models with Geodetector models to systematically investigate the determinants of hospital bed distribution across Chinese provinces. This combined approach leverages the strengths of each method: SEM accounts for spatial dependence in the residuals, thereby improving statistical validity, while Geodetector captures non-linear relationships and factor interactions without assuming linearity. Our methodology not only quantifies the optimal value ranges of key influencing factors but also reveals significant interaction effects among these determinants. Together, this integrated framework provides a more comprehensive understanding of the spatial heterogeneity in bed distribution by combining complementary analytical perspectives.

Translating these findings into actionable policy recommendations, several concrete strategies emerge. First, the significant negative correlation between sex ratio and bed availability suggests that health planning must move beyond population size and incorporate demographic structure. Regions with a disproportionately high sex ratio (i.e., a larger male population) should consider investing in specialized inpatient services for conditions with higher male prevalence, such as trauma, cardiovascular diseases, and certain occupational injuries, to better align bed supply with actual demand. Concurrently, to mitigate the risk of underutilization in other departments, health systems could implement flexible bed management strategies, including cross-departmental bed-sharing arrangements, to accommodate fluctuating demands across specialties. Second, the threshold effect observed for GDP per capita-where bed demand concentrates in lower-GDP regions-calls for a development-stage-specific policy approach. To address this threshold effect, resource allocation mechanisms could be reformed to adopt a development-stage-specific approach. For low-GDP regions, the priority should be on ensuring a sufficient baseline quantity of beds to meet essential healthcare needs, potentially through increased central government transfer payments earmarked for hospital infrastructure. Conversely, high-GDP regions should be encouraged to adopt policies that shift focus from bed quantity expansion to quality enhancement, such as promoting day surgery, strengthening primary care to reduce avoidable hospitalizations, and investing in advanced medical technologies that improve patient throughput. These regionally differentiated strategies, directly derived from our spatial evidence, offer a concrete pathway to operationalize the equity goals of the “Healthy China 2030” framework.

Several limitations should be acknowledged in this research. First, the unavailability of data for certain potential determinants may have resulted in omitted variable bias. Second, while GDP per capita was retained as the economic development indicator to align with the study's focus on bed supply, the exclusion of disposable income per capita means that household purchasing power effects on bed distribution were not assessed. Third, the provincial-level analysis may mask important sub-provincial variations, potentially limiting the precision and generalizability of our findings. These scale-related constraints suggest that more granular data collection and analysis would be valuable for future research.

## 5 Conclusion

Our analysis reveals significant spatial heterogeneity and autocorrelation in hospital bed distribution across Chinese provinces. MLR and SEM model identify a negative spillover effect of sex ratio on bed allocation, underscoring the importance of incorporating demographic structural factors in regional health resource planning. Compared to MLR, SEM demonstrates superior effectiveness in addressing spatial dependency issues.

The Geodetector analysis demonstrates that GDP per capita serves as the primary driver of bed distribution, while risk detector results uncover a threshold effect: bed demand concentrates in low-GDP regions, whereas high-GDP areas demonstrate relatively fewer hospital beds due to improved efficiency and technological advancement. This finding suggests the need for development-stage-specific health policies.

Notably, interaction effects between factors significantly amplify individual impacts, with the disposable income per capita and sex ratio interaction showing the strongest synergy. These results highlight the necessity of adopting integrated policy approaches that simultaneously address socioeconomic and demographic determinants in public health decision-making.

## Data Availability

The original contributions presented in the study are included in the article/Supplementary material, further inquiries can be directed to the corresponding author.
